# Invasive Meningococcal Disease in the Post-COVID World: Patterns of Disease Rebound

**DOI:** 10.3390/vaccines13020165

**Published:** 2025-02-08

**Authors:** Jamie Findlow, Myint Tin Tin Htar, Rodolfo Villena, Paul Balmer

**Affiliations:** 1 Pfizer Global Medical Affairs, Vaccines and Antivirals, Pfizer Ltd, Tadworth KT20 7NS, UK; 2 Pfizer Global Medical Affairs, Vaccines and Antivirals, Pfizer Inc, 75014 Paris, France; 3 Department of Pediatrics, Faculty of Medicine, Universidad de Chile, Infectious Disease Unit, Hospital de Niños Dr. Exequiel González Cortés, Santiago 8900000, Chile; rodolfo.villena@gmail.com; 4 Pfizer Global Medical Affairs, Vaccines and Antivirals, Pfizer Inc, Collegeville, PA 19426, USA

**Keywords:** invasive meningococcal disease, epidemiology, surveillance, vaccines

## Abstract

Invasive meningococcal disease (IMD) is a global health threat with an unpredictable epidemiology that varies regionally and over time. During the COVID-19 pandemic, the Invasive Respiratory Infection Surveillance Consortium reported widespread reductions in bacterial diseases transmitted via the respiratory route, including IMD, that were associated with the stringency of COVID-19 measures implemented in each country. Here, we report the epidemiology of IMD from the United States, England, France, Australia, and Chile during and after the COVID-19 pandemic. There was a consistent trend in which IMD incidence declined throughout 2020 and into 2021 but began to increase in 2021 (England and Chile) or 2022 (United States, France, and Australia). Case numbers of IMD in France and the United States surpassed pre-pandemic levels by December 2022 and 2023, respectively, whereas in other countries examined, overall cases in 2021/2022 or 2023 approached but did not exceed pre-pandemic levels. Except for the United States, meningococcal serogroup B was the prominent serogroup of post-pandemic re-emergence, although substantial increases in serogroup Y and W disease in France in 2022 and serogroup W disease in Chile in 2023 were also observed. In the United States, where meningococcal vaccination coverage did not decline during the pandemic, the rebound in cases was predominantly due to serogroups C, Y, and nongroupable serogroups. The data indicate that surveillance of IMD cases, associated serogroups, and vaccination uptake is essential for monitoring the effectiveness of disease prevention strategies and guiding future public health policy.

## 1. Introduction

Invasive meningococcal disease (IMD) caused by *Neisseria meningitidis* most commonly manifests as meningitis and/or septicemia (meningococcemia) [1]. Case fatality rates range from 4% to 20%, with a pooled estimate of 8% [2]. An estimated 10% to 20% of survivors experience long-term clinical sequelae, such as scarring, amputations, hearing and/or vision impairment, cognitive deficits, and psychological disturbances [3,4].

*N. meningitidis* is a global pathogen, and in 2019 there were an estimated 141,000 deaths due to IMD worldwide [5]. Twelve meningococcal serogroups have been identified; although the majority of disease is caused by five serogroups (A, B, C, W and Y) [6]. The epidemiology of IMD is dynamic and unpredictable, with incidence and serogroup distribution changing across different regions, age groups, and over time [6]. Understanding trends in the epidemiology of communicable diseases such as IMD is critical for informing public health policy. During the COVID-19 pandemic (2020–2021), pronounced decreases in the incidence of diseases typically caused by respiratory syncytial virus, *Streptococcus pneumoniae*, and *Haemophilus influenzae* were observed that coincided with COVID-related restrictions and containment measures [7,8,9,10]. These epidemiological shifts likely resulted primarily from the implementation of non-pharmacological interventions designed to prevent the spread of COVID-19, such as lockdowns, social distancing, quarantining, mask wearing, and improved sanitation measures [7,11]. A sharp decrease in IMD incidence was reported during the COVID-19 pandemic in 2020 in South America (Argentina, Brazil, Chile, and Uruguay) and Spain [12,13]. Significant decreases in IMD incidence during the COVID-19 lockdowns were also reported in England and France [14,15].

A concern after the pandemic and the lifting of restrictions is a rebound in disease to incidence rates similar to those before the pandemic or potentially higher [16]. It has been suggested that non-pharmaceutical interventions implemented during the pandemic could leave a higher proportion of individuals immunologically susceptible because of reduced exposure to commonly circulating microbes, so-called immunity debt, leading to future disease outbreaks [9,16]. Additionally, there was a concern that the disruption of routine vaccination schedules that occurred during the pandemic could lead to increased risk of disease [16,17]. Here, we describe the changing epidemiology of IMD during the COVID-19 pandemic and immediately following the easing of containment measures in regions with available data on IMD, including the United States, England, France, Australia, and Chile.

## 2. Methods

The epidemiology of IMD (reported number of cases and/or incidence rates) was reviewed from selected countries with established robust disease surveillance where applicable data through the end of 2023 were known to be publicly available from national surveillance system databases. Data were derived from the Centers for Disease Control and Prevention (CDC) National Notifiable Disease Surveillance System (NNDSS) [18] and the Enhanced Meningococcal Disease Surveillance [19] for the United States; the UK Health Security Agency Health Protection Reports [20] and the UK Health Security Agency for England [21]; the Santé Publique France [22] for France; the Australian Government Department of Health and Aged Care NNDSS [23] and the Australian Meningococcal Surveillance System (AMSP) [24] for Australia; and the Instituto de Salud Pública de Chile for Chile [25]. A literature search was conducted to confirm that all relevant, available data were captured. Data extracted for discussion in this review included the epidemiology of all-type IMD, serogroup-specific IMD, and related vaccination programs in the context of COVID-19 preventive measures.

## 3. Results

### 3.1. United States

In the United States, the first large scale coronavirus-related activity restrictions occurred on 16 March 2020 when a “shelter in place” order was issued for six counties in the San Francisco bay area [26]. On 19 March 2020, California issued a statewide order requiring all residents to remain at home except when engaging in essential activities, and by 3 April, 41 states had implemented statewide stay-at-home orders. Such restrictions remained in place in many states into July of 2020, when the first wave of the pandemic was mostly thought to have passed [27].

**Figure 1:**
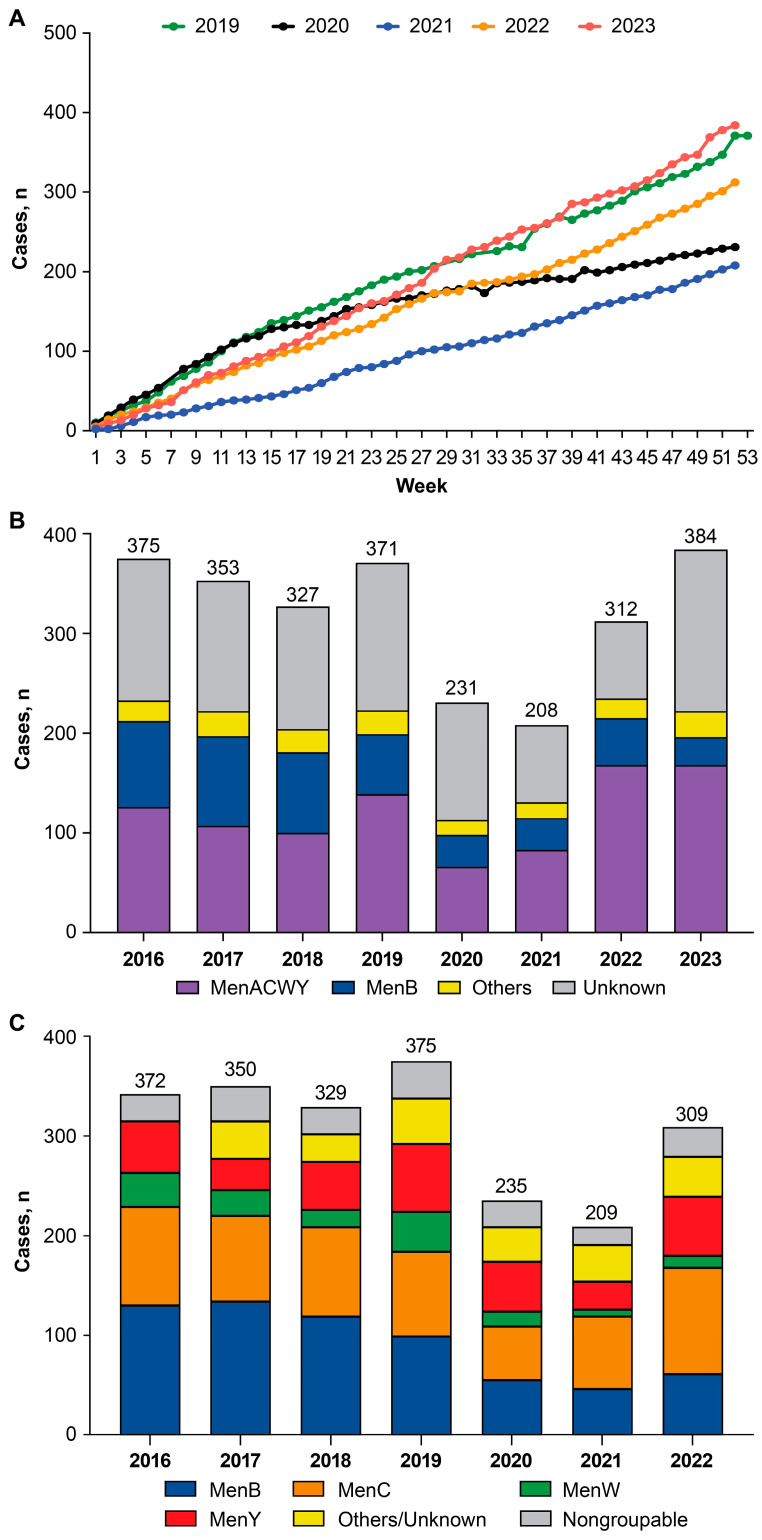
Invasive meningococcal disease cumulative case numbers (**A**) and annual case numbers by serogroup reported by the National Notifiable Diseases Surveillance System (**B**) and the Enhanced Meningococcal Disease Surveillance (**C**) in the United States: 2016–2023 (Centers for Disease Control and Prevention [18,28,29,30,31,32,33,34]). MenACWY, meningococcal serogroups A, C, W, and Y; MenB, MenC, MenW, and MenY, meningococcal serogroups B, C, W, and Y, respectively. Cumulative year-to-date and yearly case numbers are determined from periods of time when the condition was reportable in the jurisdiction (i.e., may be incomplete year-to-date data or less than 52 weeks of data).

The numbers of IMD cases reported by NNDSS in the United States were high during the years 2016–2019 (371 cases reported in 2019), declined during the COVID-19 pandemic in 2020 (231), and were lowest in 2021 (208 cases; Figure 1A,B) [18]. In 2022, 312 total cases were reported, representing an increase from the previous 2 years, and in 2023, 384 cases were reported, surpassing pre-pandemic levels (Figure 1B).

Decreases observed in overall IMD case numbers during 2020 and 2021 were predominantly reflected in decreases caused by meningococcal serogroups B (MenB), W (MenW), and nongroupable serogroups (Figure 1B,C) [18,28,29,30,31,32,33,34]. As reported by the EMDS, the post-COVID-19 pandemic increases in IMD cases in 2022 were caused predominantly by serogroups C (73 cases in 2021 vs. 107 cases in 2022), Y (28 cases in 2021 vs. 59 cases in 2022), and nongroupable serogroups (18 cases in 2021 vs. 30 cases in 2022). As reported by NNDSS, IMD cases caused by MenACWY and unknown serogroups exceeded pre-pandemic levels in 2023 (MenACWY: 139 cases in 2019 and 168 cases in 2023; unknown serogroups: 148 cases in 2019 and 162 cases in 2023; note that serogroup A is very rare in the United States [35]). However, isolates for 2023 are still being processed, and more accurate serogrouping data for the currently unknown serogroups will be released with the 2023 EMDS report. In March 2024, the CDC issued a health advisory alert for an increase in serogroup Y meningococcal disease [36]. Furthermore, in February 2024 the CDC reported an increase in antibiotic resistance serogroup Y isolates [37]. There were 148 cases of serogroup Y disease reported in 2023 and of 94 patients with known outcomes, 17 (18%) died; a case-fatality rate higher than the historical case-fatality rate of 11% for serogroup Y. A substantial proportion of IMD cases in the US were nongroupable (Figure 1C), which was not mirrored in the other countries (see below) where few, if any, cases of disease were caused by nongroupable serogroups.

The CDC recommends adolescents 11 to 12 years of age receive a MenACWY vaccine, with a booster dose at 16 years, and shared decision-making is recommended for adolescents and young adults to determine if the MenB vaccine is appropriate [38]. If a patient is receiving MenACWY and MenB vaccines at the same visit, pentavalent MenABCWY may be given instead (Table 1) [38]. Meningococcal vaccine coverage rates during the pandemic, compared with pre-pandemic rates, were slightly higher for MenB but remained similar for MenACWY. Estimated adolescent (17 years of age) coverage rates for receipt of at least one dose of MenB vaccine were 14.5% (2017), 17.2% (2018), 21.8% (2019), 28.4% (2020), 31.4% (2021), and 29.4% (2022) [39,40,41]. The estimated adolescent (13–17 years of age) coverage rates for receipt of at least one dose of MenACWY vaccine were 85.1% (2017), 86.6% (2018), 88.9% (2019), 89.3% (2020), 89.0% (2021), and 88.6% (2022).

Two outbreaks of meningococcal disease have been reported in the United States since 2020. A serogroup C outbreak among gay and bisexual men since December of 2021 in the state of Florida has been linked to at least 24 cases and 6 deaths [69,70]. The number of cases of meningococcal disease reported in Florida through August 2022 was higher (50 cases) than that reported through August in each of the 5 previous years (17–27 cases), likely attributable to the serogroup C outbreak [71]. In Eastern Virginia, 12 cases of serogroup Y IMD were reported between June 2022 and 8 March 2023, resulting in 3 deaths [72]. Affected individuals were mostly Black adults 30 to 60 years of age; 11 were not vaccinated against meningococcal serogroup Y, and 1 was partially vaccinated (i.e., had not received all doses of the vaccine).

### 3.2. England

In response to COVID-19, the UK government implemented a stringent lockdown on 23 March 2020, declaring no person may leave the place where they are living without reasonable excuse, including closure of most schools, playgrounds, leisure facilities, and gyms [73]. Lockdown measures were eased in July of 2020; however, subsequent national lockdowns were implemented in November 2020 and January 2021.

**Figure 2:**
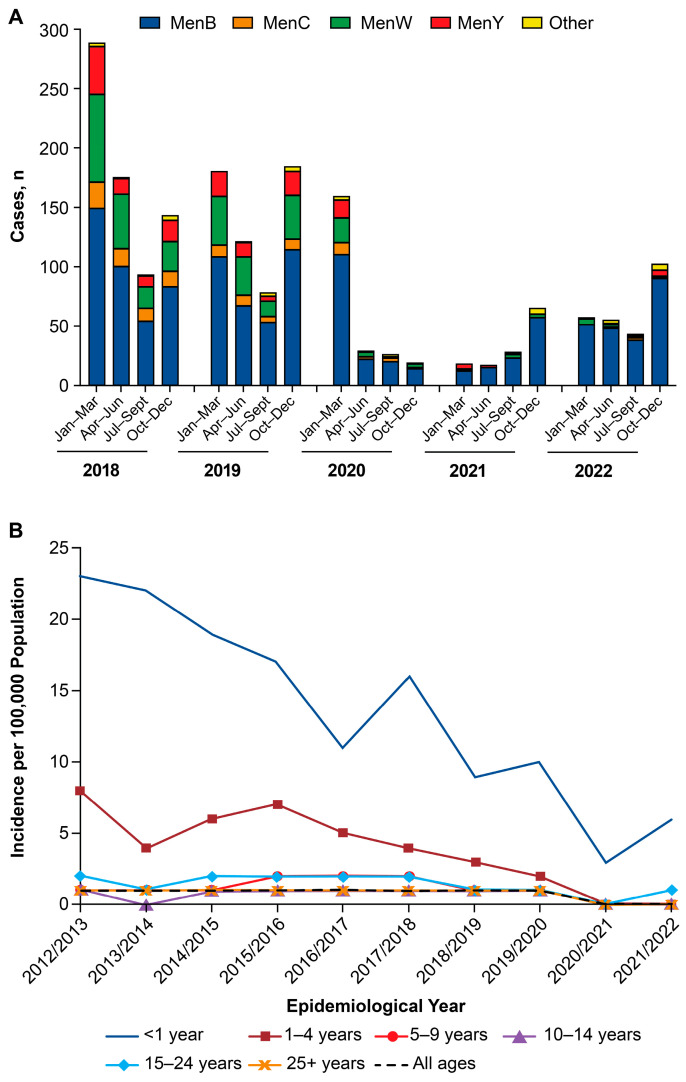
Quarterly invasive meningococcal disease case numbers by serogroup (**A**) and yearly incidence by age group (**B**) in England: 2018–2022 ([43,44,45,46,47,48,49,50,51,52,53,74,75]; contains public sector information licensed under the Open Government License v3.0). MenB, MenC, MenW, and MenY, meningococcal serogroups B, C, W, and Y, respectively; Other, serogroups A, X, E, Z, or ungroupable and ungrouped serogroups.

Case numbers in England were obtained from the UK Health Security Agency, which details the annual incidence of IMD overall and by age and serogroup [20]. Cases of IMD decreased substantially beginning in the second quarter of 2020 after the start of the pandemic, with low case numbers continuing through the first half of 2021 (Figure 2A) [43,44,45,46,47,48]. Cases began to increase in the second half to 2021 through 2022, with 102 cases of IMD in the fourth quarter of 2022 [44,49,50,51,52,53,74]. Cases of IMD during the pandemic, as well as the rebound cases in 2021/2022, were mostly MenB (Figure 2A). The proportion of cases attributable to the CWY serogroups during the pandemic and during the rebound were near or below the proportion attributable to these serogroups before the pandemic (i.e., approximately 30%). Before the pandemic, IMD incidence was highest in infants and young children <5 years of age in England, and the decrease in IMD incidence during the pandemic (epidemiologic years 2019/2020 and 2020/2021) was mostly due to decreased IMD in these age groups (Figure 2B); although substantial decreases in IMD cases also occurred in those over 25 years of age (Supplementary Figure S1). The increased incidence following the relaxation of pandemic control measures occurred mostly in infants <1 year of age and young adults 15 to 24 years of age (Figure 2B and Supplementary Figure S1) [75].

In the United Kingdom, the National Health Service recommends MenB vaccination at 8 weeks, 16 weeks, and 1 year of age, and the MenACWY vaccine is offered to teenagers at 14 years of age (school years 9 and 10; Table 1) and those up to 25 years of age who are MenC vaccine-naïve [42]. The COVID-19 pandemic had little effect on the rates of MenB vaccine uptake; however, MenACWY vaccine uptake decreased in year 9 students in the 2019–2020 academic year and in year 10 students in the 2020–2021 academic year, remaining lower through the 2021–2022 academic year (Table 2) [76,77].

### 3.3. France

In response to the COVID-19 pandemic, French public health authorities issued recommendations for preventing COVID-19 infection in March of 2020. The government implemented a national lockdown on 17 March 2020, with a partial easing of restrictions on 11 May 2020, and full removal of restrictions in June 2020 [78]. Under the lockdown, people could go outside for necessary shopping, for physical activity, and to help vulnerable populations. Only those whose jobs were deemed essential were allowed out of their homes to go to work.

**Figure 3:**
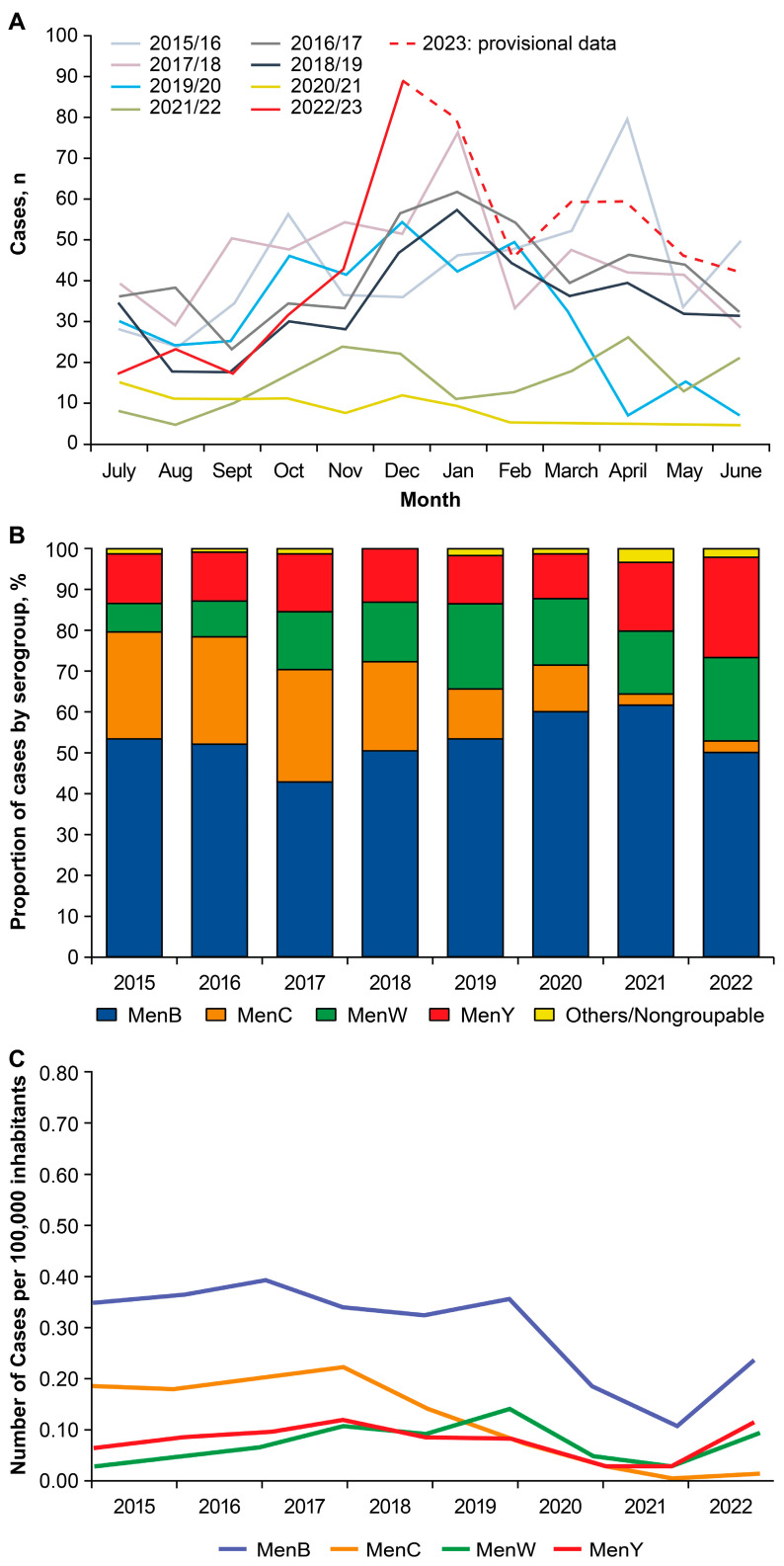
Yearly cases of invasive meningococcal disease by month (**A**), by proportion of each serogroup (**B**), and incidence by serogroup (**C**) in France: 2015–2023 [22].

Analysis of data from the French National Reference Center for Meningococci and *H. influenzae* (NRCMHi) found that substantial decreases in overall IMD case numbers were observed in 2020 and 2021 and continued through the first half of 2022 (Figure 3A) [22]. Case numbers of IMD started to increase beginning in the second half of 2022, worsened through the winter season, and surpassed monthly pre-pandemic levels by December of 2022.

Except for 2017, MenB was the predominant serogroup from 2015 through 2022, accounting for about 50% to 60% of all cases occurring during this period (Figure 3B,C) [22]. During and after the pandemic, from 2021 through to 2022, the proportion of IMD cases attributable to MenC sharply decreased, while the proportion of cases attributable to MenW and MenY increased (Figure 3B). The rebound in IMD in 2022 occurred in MenB, MenW, and MenY (Figure 3C). Of the 314 IMD cases for which the serogroup was characterized in 2022, 158 (50.3%) were MenB, 77 (24.5%) were MenY, 64 (20.4%) were MenW, 8 (2.5%) were MenC, and 7 (2.2%) were linked to nongroupable strains [22]. The increases in IMD occurred most prominently in age groups 15–24 years, 25–59 years, and ≥60 years for MenY and MenW, and age groups 15–24 years and 25–59 years for MenB (Supplementary Figure S2) [22]. The increased cases of MenY surpassed pre-pandemic (i.e., 2019) MenY case numbers and mostly occurred in those aged 15 years and older.

Deghmane et al. [79] performed whole genome sequencing on 1466 IMD isolates obtained by the national reference center for meningococci and *Haemophilus influenzae* (NRCMHi) in France from 2017 to 2021. Relative to the pre-pandemic years (2017–2019), the proportion of isolates due to hyperinvasive clonal complexes (CCs) in 2020 (mostly CC11 and CC269) decreased, whereas, in 2021, the proportion began to increase, predominantly because of increases in hyperinvasive CC269 and CC32. Recently, analysis of data between 2015 and 2022 from the French National Reference Center Database for meningococci demonstrated a decline in the number of IMD cases across all serogroups and age groups in 2020 and 2021 [80]. The decline in cases from 2019 through 2021 was predominantly due to decreases in hyperinvasive CC11 [80]. Since 2021, it has been reported that IMD cases increased across all age groups, predominantly due to serogroup B CC32, CC41/44, other/non-assigned isolates, serogroup W CC9316, and all genotypes of serogroup Y.

In France, MenC vaccination was mandatory at 5 months of age, with a possible catch-up for individuals up to 24 years of age, and MenB vaccination was recently recommended for those 2 months to 2 years of age (two vaccinations and one booster; Table 1) [22,54]. Public Health France reported that the coverage rates for receiving at least one dose of the MenC vaccine by 21 months of age were 77.2% (2017), 82.3% (2018), 85.1% (2019), 87.6% (2020), 87.5% (2021), and 87.0% (2022) [81]. Taine et al. [82] did report that the rate of uptake of MenC primary and booster doses in France declined during the first 10 months of the pandemic. Vaccination of infants against MenB (two vaccinations and one booster; Table 1) in France has been recommended since June 2022 [81]. Coverage for at least one dose of the MenB vaccine at 8 months of age was 48.8% in 2022 and 74.7 in 2023; receipt of three doses by 21 months of age was 35.1% in 2022 [81].

Two outbreaks of MenB have been reported in France since the COVID-19 pandemic. Between August 2021 and July 2022, 12 MenB cases were reported in southeast France, 11 of them among adolescents/young adults ages 16 to 21 years [22]. In December of 2022, the French public health authorities reported a cluster of four MenB cases in young adults who had visited the same nightclub between 1 November and 28 November 2022 [83]. One death was reported among the four cases.

### 3.4. Australia

In Australia, a nationwide partial lockdown was implemented near the end of March 2020, which included closures of many non-essential businesses, advisories to work from home where possible, and a transition to online learning in schools and universities [84]. The lockdown ended in early May of 2020, although some measures, such as mandatory mask-wearing in some settings and restrictions on interstate travel, remained in place. However, Victoria, the second most populous state, implemented a more stringent lockdown during the second half of 2020 that lasted until late October 2020.

**Figure 4:**
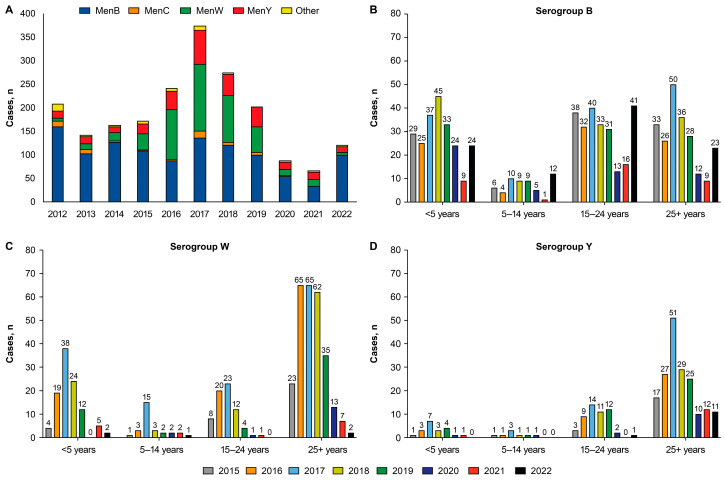
Annual invasive meningococcal disease case numbers by serogroup (**A**) and age (**B**–**D**) in Australia through 2022 [23,58,59,60,61,62,63,64,65,66]. MenB, meningococcal serogroup B; MenC, meningococcal serogroup C; MenW, meningococcal serogroup W; MenY, meningococcal serogroup Y; Other, non-BCWY serogroups.

Australia’s NNDSS reports cases of probable and laboratory-confirmed IMD [23,60]. The National Neisseria Network is a collaborative network of reference laboratories that coordinates laboratory data from IMD cases for the Australian Meningococcal Surveillance Program; these data supplement the notification data from the NNDSS (e.g., with serogroup data) [23,60]. Numbers of reported IMD cases peaked at 374 in 2017 and gradually fell to 67 cases in 2021 (Figure 4A) [23,58,59,60,61,62,63,64,65,66]. A decline in IMD notifications was observed following substitution of the monovalent MenC vaccine with the quadrivalent MenACWY vaccine in Australia’s National Immunization Program in 2018 [60]. After the introduction of the quadrivalent vaccine, MenB became the predominant disease-causing serogroup, accounting for 50% of cases in 2019 (Figure 4A). Before the pandemic (2017–2019), IMD was mostly due to MenB, MenW, and MenY (Figure 4A). During this pre-pandemic period, MenB predominantly occurred in children <5 years of age and those ≥15 years of age; MenW predominantly occurred in adults ≥25 years of age, with smaller peaks in children <5 years and those 15 to 24 years of age; and MenY predominantly occurred in adults ≥25 years of age, with a smaller peak in those 15 to 24 years of age (Figure 4B–D). Case counts declined in 2020 and 2021, mostly due to declines in cases involving serotypes B, W, and Y, and started to rebound in 2022 (Figure 4A). The rebound in IMD cases in 2022 was entirely due to MenB, which occurred across all age groups (Figure 4A,B).

In Australia, MenACWY is recommended for children aged 12 months and adolescents aged 14–16 years, and MenB is recommended for Aboriginal and Torres Strait Islander children aged 2, 4, 6, and 12 months (Table 1) [55]. Additionally, the states of Queensland and South Australia have vaccination programs that offer the MenB vaccine to infants 6 weeks to 12 months of age and adolescents 15–19 years of age (Queensland) and in school Year 10 (South Australia) [56,57]. Coverage of MenACWY vaccines in adolescents by 17 years of age in Australia was 74.3% in 2020, 76.1% in 2021, and 75.9% in 2022 [85,86]. Coverage of MenB vaccine for indigenous children was 78.7%, 75.8%, and 63.8% in 2021, and 80.4%, 78.6%, and 69.8% in 2022 for the first, second, and third doses, respectively (note funding for MenB vaccination for indigenous children was initiated in July 2020) [85,86].

### 3.5. Chile

In response to the COVID-19 pandemic, Chile implemented non-pharmaceutical interventions (NPIs) such as stay-at-home orders and travel restrictions at the municipal level [87]. At a country level, initial NPIs were implemented in mid-March 2020 and included school closings, banning of public gatherings, and mandatory self-isolation of passengers traveling from high-risk countries and mandatory use of masks, even for outdoors activities [88]. Following a surge in COVID-19 cases, a lockdown was implemented in different regions, starting with the entire Santiago metropolitan region on 15 May [88,89].

**Figure 5:**
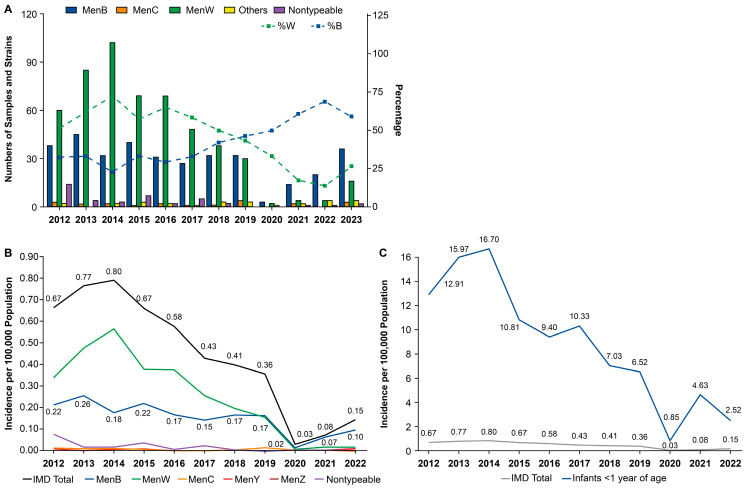
Number of cases (samples and strains) of confirmed IMD (**A**) and incidence of IMD by serogroup (**B**) and incidence of IMD in infants <1 year of age (**C**) in Chile: 2012–2023 [25]. IMD, invasive meningococcal disease; MenB, meningococcal serogroup B; MenC, meningococcal serogroup C; MenW, meningococcal serogroup W; MenY, meningococcal serogroup Y; Others, non-BCWY serogroups.

In Chile, the number of laboratory-confirmed samples, and incidence, of IMD decreased substantially among all serogroups from before the pandemic to 2020 (Figure 5A,B) [25]. Beginning in 2021, a re-emergence was observed, most prominently with MenB and to a lesser extent MenW, whereas the incidence of IMD caused by other serogroups remained very low. The number of MenW IMD cases predominated from 2012–2018, whereas MenB was the most common serogroup from 2019–2023, accounting for 59% of cases in 2023. IMD incidence was highest in infants <1 year of age and generally followed the trends in IMD incidence in the total population from 2012 to 2022 (Figure 5B,C). The rate of IMD was highest among infants <1 year of age in 2021 (11 cases), 2022 (6 cases), and 2023 (14 cases) [25].

The Ministry of Health in Chile recommends a MenACWY vaccine at 12 months of age and in July 2023 incorporated a MenB vaccine at 2 and 4 months of age with a booster dose during the second year of life into the immunization program (Table 1) [67,90]. Meningococcal vaccination coverage in Chile for MenACWY had been high in the 2015–2019 period (median: 97%), with a slight decrease during and after the COVID-19 pandemic years; vaccination coverage in the target population (12 months old) was 94.5% (2019), 91.9% (2020), 89.6% (2021), 89.5% (2022), and 92.8% (2023) [91,92,93,94]. For the MenB vaccine, coverage in 2023 was 98.7% for dose one and 96.5% for dose two [94].

## 4. Discussion

During the COVID-19 pandemic, substantial worldwide decreases in diseases typically transmitted via the respiratory route, such as *S. pneumoniae, H. influenzae*, and *N. meningitidis* were observed, likely due to the implementation of non-pharmacological interventions designed to prevent the spread of COVID-19 [16]. Some predicted that meningococcal disease would remain low and not rebound. In five countries where data are publicly available regarding IMD case numbers during and after the pandemic, we found that a consistent trend emerged in which the incidence of IMD declined throughout 2020 and into 2021 but began to increase in 2021 (England and Chile) or 2022 (United States, France, and Australia) [18,22,23,43,44,45,46,47,48,49,50,51,52,53,58,59,60,61,62,63,64,65,66,68,74]. Case numbers of IMD in France surpassed pre-pandemic levels by December 2022 and in the United States in the latter half of 2023, whereas in other countries examined, overall case levels in 2021/2022 approached but did not exceed pre-pandemic levels. It is possible that reductions in reported cases of IMD resulted from disruption of routine invasive disease surveillance while countries were responding to the COVID-19 pandemic. However, the Invasive Respiratory Infection Surveillance Initiative, which consists of a network of reference laboratories in 26 countries and territories, reported that they did not observe disruptions in routine submissions of *S. pneumoniae*, *H. influenzae*, *N. meningitidis*, or *S. agalactiae* to the reference laboratories in 2020 compared with 2018 and 2019 [7].

In 2020, routine vaccination rates fell worldwide [95] due to vaccination programs being temporarily suspended, lockdown policies, vaccine supply, or administration issues [96]. In a survey of 4962 parents from eight countries (United States, United Kingdom, Italy, France, Germany, Argentina, Brazil, and Australia) in 2021, 50% reported delaying or forgoing meningococcal vaccinations [82,97]. In the current analysis, meningococcal vaccination coverage rates were found to decrease during the pandemic in England (MenACWY vaccination), and Chile, but did not decline in the Unites States or France (MenC vaccination). Declining vaccination and booster rates may have left many individuals unprotected or only partially protected, respectively, contributing to an increased risk of meningococcal outbreaks and overall disease burden once COVID-related restrictions and containment measures were eased. Additionally, low exposure to environmental pathogens due to lockdown measures, such as quarantining and school/business closures, may have resulted in a relative lack of immune stimulation, inducing an “immunity debt” that potentially left the pediatric population in particular more susceptible to infection once restrictions were lifted [9].

The COVID containment measures may have resulted in a decline in asymptomatic carriage of *N. meningitidis* or closely related species, such as *Neisseria lactamica,* which have been shown to confer protection against meningococcal disease (via induction of cross-reactive adaptive immune responses in the case of *N. lactamica*), potentially contributing to a decline in herd protection in the general population [9,98,99]. Despite decreases in IMD cases in Australia during the pandemic, the overall meningococcal pharyngeal carriage prevalence in adolescents and young adults aged 17 to 25 years in South Australia in 2018–2020 was not reduced by the public health strategies put in place to reduce COVID-19 transmission, thus, suggesting that carriage continued despite mitigation measures [100]. Pharyngeal carriage of disease-associated meningococci in adolescents and young adults in South Australia was found to be significantly higher during the COVID-19 period versus the pre-COVID era (6.8% vs. 3.7%; *p* = 0.01), mainly driven by increases in MenB and MenY, despite the increased uptake of 4CMenB and MenACWY vaccines during the COVID-19 period. However, nongroupable meningococcal carriage (i.e., positive for the meningococcal *porA* gene but negative for capsular genes for disease-causing groups A, B, C, W, X, or Y) did decrease significantly during COVID-19 compared with the pre-COVID era (from 3.8% to 1.7%; *p* = 0.04).

In the current evaluation, with the exception of the United States, serogroup B was the most prominent serogroup of re-emergence, and in Australia, case numbers of MenB in 2022 were similar to pre-pandemic (i.e., 2019) case numbers. In England, France, and Australia, countries that recommend MenB vaccination only for infants and toddlers, the rebound in serogroup B cases mostly occurred in adolescents and young adults. In the United States, where meningococcal vaccination coverage did not decline during the pandemic and MenB vaccination in adolescents slightly increased, the rebound in cases occurred across multiple serogroups, including serogroups C, W, and Y, as well as nongroupable serogroups. In countries where MenACWY vaccines were previously introduced, such as England, Australia, and Chile, serogroups C, W, and Y IMD cases remained relatively low during the rebound, although substantial increases in MenW were observed in Chile in 2023. In France, where MenACWY vaccines are not routinely recommended, the re-emergence of W and Y serogroups, in addition to B, were observed in adolescents, young adults, and older adults. The findings suggest the importance of MenACWY vaccination programs to reduce disease burden caused by these serogroups. Recently, Hadley et al. [101] adapted a mathematical model for meningococcal carriage and disease to predict the impact of the adolescent MenACWY vaccination program in the United Kingdom on pre-pandemic meningococcal transmission and the effect that social distancing and reduced vaccine uptake may have on the future epidemiology of meningococcal carriage and disease. The model demonstrated that the MenACWY vaccine program resulted in indirect protection from, and suppression of, transmission by 2020. It also predicted that COVID-19 social distancing was expected to have accelerated the declines in transmission, resulting in significant long-term reductions in carriage prevalence of serogroups A, C, W, and Y and incidence of invasive disease. These model estimates are consistent with findings from countries in the current evaluation that have toddler or adolescent MenC or MenACWY vaccination programs, which had few, if any, rebound cases of MenC, MenW, and MenY. Global variations in recent MenW disease epidemiology underscore the importance of proactive vaccination strategies. Many countries (e.g., Chile, Australia, United Kingdom, the Netherlands) implemented MenACWY vaccination programs only after experiencing major MenW disease increases [6,102,103]. However, the United States, which has recommended the MenACWY conjugate vaccine for adolescents since 2005, has not experienced a significant increase in MenW disease [6].

The World Health Organization has developed a global roadmap to defeat meningitis by 2030, with achievement of higher vaccination coverage in the population as one of the main goals [104]. Our findings suggest the occurrence of a post-pandemic rebound in meningococcal disease globally, particularly in countries without active meningococcal vaccination programs. An evaluation of trends in invasive bacterial diseases across 30 countries and territories in the Invasive Respiratory Infection Surveillance Consortium indicated that, following decreases during the pandemic, cases of meningococcal disease were increasing by the end of 2021 [16]. The disruption in immunizations during the COVID-19 pandemic and the phenomenon of “immunity debt” likely contributed to reductions in herd protection and increased the number of individuals susceptible to IMD [9]. Accordingly, disease outbreaks and a rebound in IMD cases have been observed following the easing of restrictions. Catch-up vaccination programs and boost vaccination strategies were previously recommended to prevent disease rebound [9,105,106] and remain critical steps to counter the factors driving the rebound, and reduce further IMD-related morbidity and mortality. Vaccination strategies should consider the predominant causal serogroup(s) during the rebound to ensure targeted coverage moving forward; for example, incorporating vaccination against MenB into national immunization programs when absent. 

## 5. Conclusions

While post-pandemic rebounds in IMD have been observed, with rebounds in case numbers greater than pre-pandemic levels in some countries, continued and expanded surveillance of IMD cases and associated serogroups will be important to understand post-pandemic meningococcal epidemiology and whether cases will continue to increase or, as recently reported in England, whether cases will return to a more stable dynamic with annual fluctuations [107]. Understanding the epidemiology of vaccination uptake will be essential for monitoring the effectiveness of disease prevention strategies and guiding future public health policy.

## Figures and Tables

**Table 1:** Summary of MenACWY and MenB vaccine programs and pre- vs. post-COVID-19 * meningococcal disease rates by country.

Country	Vaccine Program	Summary of Pre vs. Post COVID-19 Evolution of IMD
United States	MenACWY—Adolescents 11–12 years of age should receive, with a booster at age 16 years [38]. MenB—For adolescents and young adults, shared decision-making is recommended for adolescents and young adults to determine if the MenB vaccine is appropriate [38]. If a patient is receiving MenACWY and MenB vaccines at the same visit, pentavalent MenABCWY may be given instead [38].	Decreases in IMD in 2020 and 2021 across all serogroups. IMD increased in 2022, predominantly due to unknown serogroups, with increases remaining under 2019 case numbers but may potentially exceed 2019 case numbers in 2023. In 2022, cases were predominantly due to unknown serogroups, followed by MenACWY, then MenB [18].
England	MenACWY—Offered to teenagers at 14 years of age and those up to 25 years of age who have not had a vaccine containing MenC [42]. MenB—Recommended for infants at 8 weeks of age with a second dose at 16 weeks and booster at 1 year [42]. MenC—Offered at 1 year of age as a combined Hib/MenC vaccine [42].	IMD cases decreased in the second quarter of 2020, across all serogroups, and rebounded in the last quarter of 2021. Increased IMD in 2022 was predominantly due to MenB in adolescents/young adults. MenACWY cases remained very low from second quarter of 2020 and onwards [43,44,45,46,47,48,49,50,51,52,53].
France	MenC—Mandatory at 5 months of age with a possible catch-up for individuals up to 24 years [22,54]. MenB—Recommended for those 2 months to 2 years of age (two vaccinations and one booster) [22,54]. MenACWY—Recommended for those with risk factors [22,54]. Compulsory vaccination of infants starting 1 January, 2025 [54].	IMD cases decreased in 2020–2021, then increased in 2022. Increased cases in 2022 occurred in MenB, MenW, and MenY. Increases in MenB in 2022 occurred predominantly in adolescents/young adults. MenY cases in 2022 exceeded those in 2019, particularly in adolescents/young adults. Increases in MenW cases in 2022 occurred predominantly in infants, adolescents, young adults, and older adults [22].
Australia	MenACWY—Recommended for children aged 12 months of age and adolescents 14–16 years [55]. MenB—Recommended for Aboriginal and Torres Strait Islander children aged 2, 4, 6, and 12 months [55]. MenB—In states of Queensland and South Australia offer to infants 6 weeks to 12 months of age and adolescents 15 to <20 years of age (Queensland) and in school Year 10 (South Australia) [56,57].	IMD cases decreased in 2020–2021 then increased in 2022, exceeding case numbers from 2020. Increases in 2022 were due to increases in MenB, which reached approximately the same level as in 2019, while cases of MenACWY remained low [23,58,59,60,61,62,63,64,65,66].
Chile	MenACWY—Recommended at 12 months of age [67]. MenB—Recommended for infants at 2 and 4 months of age with a booster dose during the second year of life [67].	IMD decreased substantially in 2020. IMD rebounded in 2021 and 2022, mostly due to increases in MenB, with MenACWY remaining low [68].

IMD, invasive meningococcal disease; MenACWY, meningococcal serogroups ACWY; MenB, meningococcal serogroup B. * Pre-COVID-19 is before 2020 and post-COVID-19 is 2020 and later.

**Table 2:** MenB and MenACWY meningococcal vaccine coverage rates in England: 2017–2022 [76,77].

Year	Vaccine Coverage Rates, %			
	MenB *	MenB Booster ^†^	MenACWY Year 9 Students	MenACWY Year 10 Students
2017–2018	92.5	N/A	86.2	84.6
2018–2019	92.0	87.8	88.0	86.7
2019–2020	92.5	88.7	58.3	87.0
2020–2021	92.1	89.0	76.3	80.8
2021–2022	N/A	N/A	69.2	79.6

IMD, invasive meningococcal disease; MenACWY, meningococcal serogroups ACWY; MenB, meningococcal serogroup B; N/A = not available. * Vaccinated by the first birthday; ^†^ Vaccinated by the second birthday.

## Data Availability

This article is based on published literature and, therefore, does not contain any applicable data sets.

## Supplementary Materials

The following supporting information can be downloaded at: https://www.mdpi.com/article/10.3390/vaccines13020165/s1, Supplementary Figure S1. Invasive meningococcal cases of serogroup B (A), serogroup C (B) and serogroup W (C) and serogroup Y (D) disease by age group in England: Epidemiological years 2012/2013–2021/2022. Supplementary Figure S2. Yearly cases of invasive meningococcal disease by age group for serogroup B (A) serogroup C (B) serogroup W (C), and serogroup Y (D) in France from 2016 to 2022.
