# Supplementary material for: Invasive Meningococcal Disease in the Post-COVID World: Patterns of Disease Rebound
Source: Vaccines (Basel). 2025 Feb 8;13(2):165. doi: 10.3390/vaccines13020165 (PMC11861802; doi:10.3390/vaccines13020165)
Supplement: Supplementary file 1 [file vaccines-13-00165-s001.zip › vaccines-3255274-supplementary.pdf]

Supplementary Materials

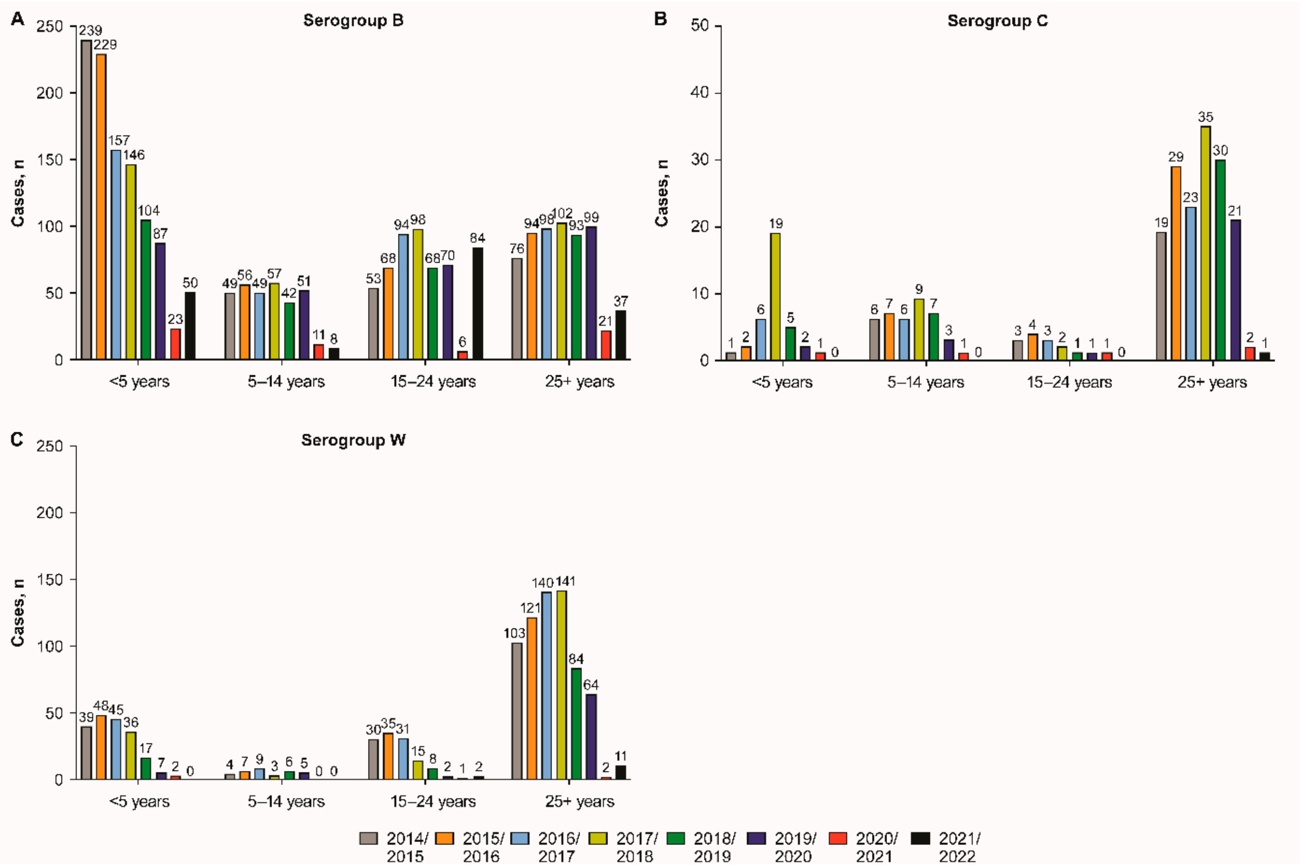

Supplementary Figure S1. Invasive meningococcal cases of serogroup B (A), serogroup C (B) and serogroup W (C) and serogroup Y (D) disease by age group in England: Epidemiological years 2012/2013–2021/2022. (UK Health Security Agency. Invasive meningococcal disease in England: annual laboratory confirmed reports for epidemiological year 2021 to 2022).

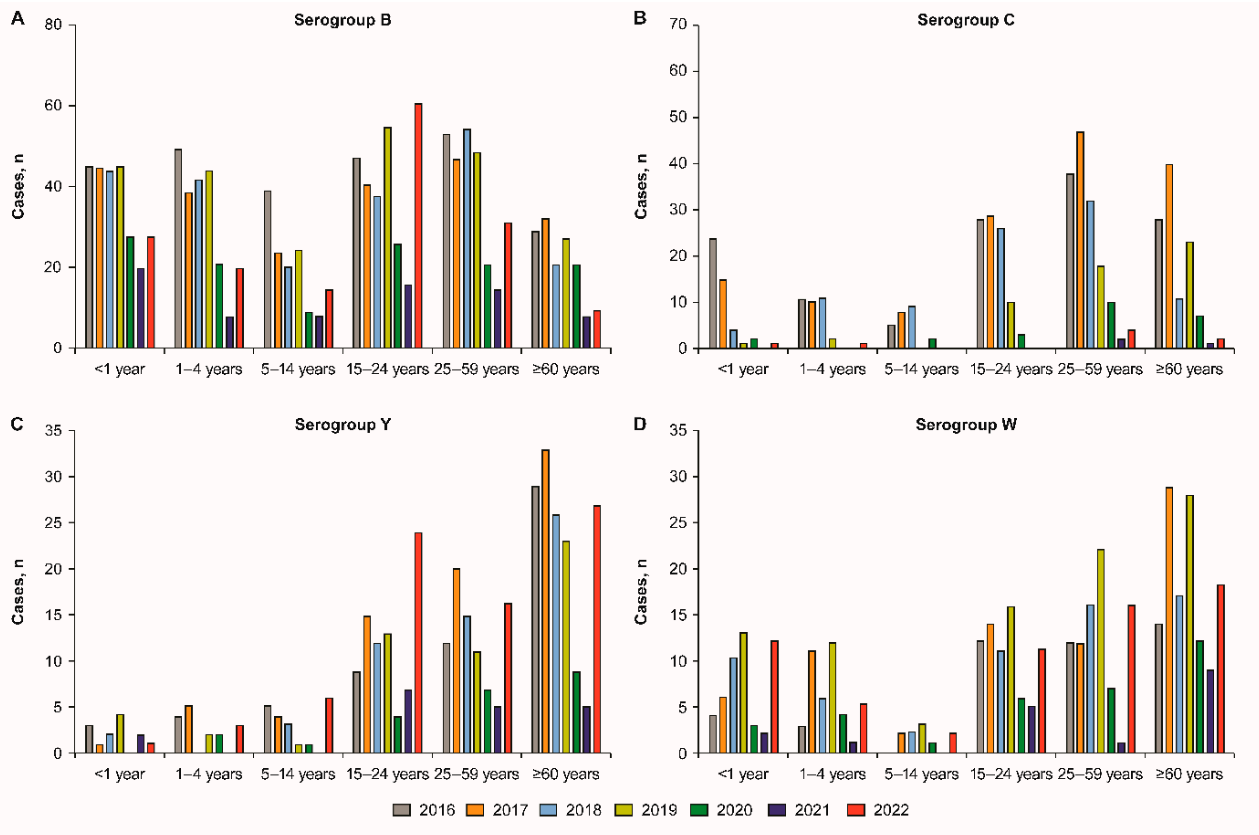

Supplementary Figure S2. Yearly cases of invasive meningococcal disease by age group for serogroup B (A) serogroup C (B) serogroup W (C), and serogroup Y (D) in France from 2016 to 2022. (Santé Publique France. Invasive meningococcal disease in France in 2022).
